# Distinct mechanisms of signal processing by lamina I spino-parabrachial neurons

**DOI:** 10.1038/s41598-019-55462-7

**Published:** 2019-12-17

**Authors:** K. Agashkov, V. Krotov, M. Krasniakova, D. Shevchuk, Y. Andrianov, Y. Zabenko, B. V. Safronov, N. Voitenko, P. Belan

**Affiliations:** 1 Department of Molecular Biophysics, Bogomoletz Institute of Physiology, Kyiv, 01024 Ukraine; 2 Department of Sensory Signaling, Bogomoletz Institute of Physiology, Kyiv, 01024 Ukraine; 3 Instituto de Investigação e Inovação em Saúde, Universidade do Porto, Porto, Portugal; 4 Neuronal Networks Group, Instituto de Biologia Molecular e Celular (IBMC), Universidade do Porto, Porto, 4200-135 Portugal; 5 Kyiv Academic University, Kyiv, 03142 Ukraine

**Keywords:** Cellular neuroscience, Pain

## Abstract

Lamina I spino-parabrachial neurons (SPNs) receive peripheral nociceptive input, process it and transmit to the supraspinal centres. Although responses of SPNs to cutaneous receptive field stimulations have been intensively studied, the mechanisms of signal processing in these neurons are poorly understood. Therefore, we used an *ex-vivo* spinal cord preparation to examine synaptic and cellular mechanisms determining specific input-output characteristics of the neurons. The vast majority of the SPNs received a few direct nociceptive C-fiber inputs and generated one spike in response to saturating afferent stimulation, thus functioning as simple transducers of painful stimulus. However, 69% of afferent stimulation-induced action potentials in the entire SPN population originated from a small fraction (19%) of high-output neurons. These neurons received a larger number of direct Aδ- and C-fiber inputs, generated intrinsic bursts and efficiently integrated a local network activity via NMDA-receptor-dependent mechanisms. The high-output SPNs amplified and integrated the nociceptive input gradually encoding its intensity into the number of generated spikes. Thus, different mechanisms of signal processing allow lamina I SPNs to play distinct roles in nociception.

## Introduction

Spinal lamina I projection neurons play an important role in nociception relaying peripheral Aδ- and C-afferent inputs to the supraspinal centres generating sensation of pain. Their ascending axons target specific brainstem and thalamic nuclei^1–5^, which further project to the nociceptive processing areas in the amygdala, hypothalamus and primary somatosensory cortex^3,6,7^. Thus, the lamina I projection neurons give rise to the ascending pathways transmitting emotional as well as discriminative aspects of pain.

A vast majority of lamina I projection neurons target the lateral parabrachial area in the brainstem^1^, being spino-parabrachial neurons (SPNs). Responses of SPNs to noxious stimulation of cutaneous receptive fields were intensively studied *in vivo* by extracellular recordings, which showed diverse patterns of their output activity^8–12^. However, these studies did not focus on the mechanisms determining diversity of specific input-output characteristics of the SPNs. Furthermore, it is still not clear how the SPNs, major output elements of the spinal network, encode noxious inputs. This knowledge is, however, necessary for better understanding both the physiology of nociception and pathology of pain syndromes.

Here we did the tight-seal recordings of primary afferent inputs to the lamina I SPNs in the intact spinal cord preparation. We revealed distinct groups of SPNs according to their input-output characteristics, which were determined by specific combinations of intrinsic firing properties, primary afferent inputs and degree of interaction with a local neuronal circuitry. These mechanisms were found to play a crucial role in the output signal encoding by SPNs. Thus, our study revealed synaptic and cellular mechanisms, which allow lamina I SPNs to play different roles in processing of nociceptive information.

## Results

### Types of SPNs

**Figure 1:**
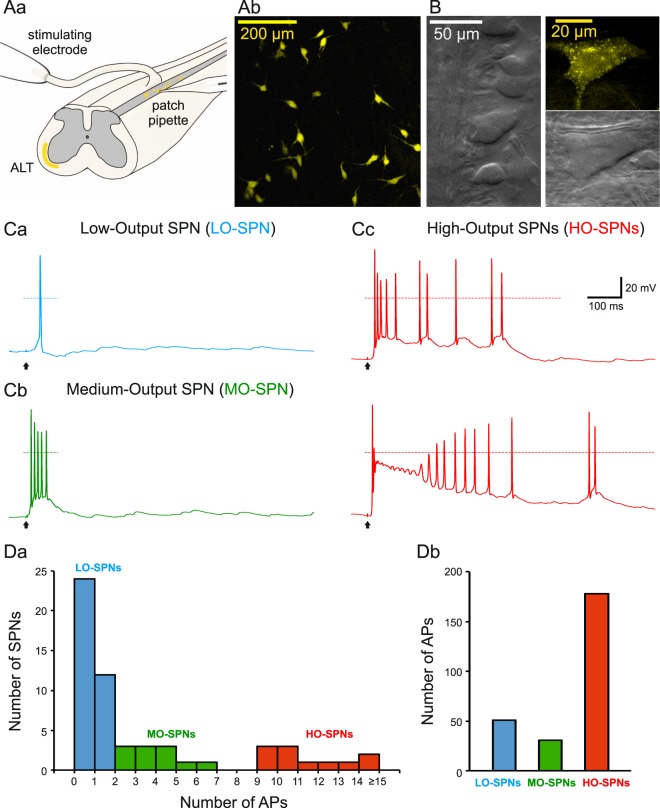
Diversity of lamina I spino-parabrachial neurons (SPNs). (**Aa**), experimental design. The whole-cell patch-clamp recordings were made from the retrogradely-labeled lamina I SPNs in the intact spinal cord preparation. ALT, anterolateral tract. (**Ab**), confocal images (Z-stack, 30 µm, at a 3 µm step) of labeled lumbar SPNs. (**B**), (left) lamina I neurons viewed in an *ex-vivo* spinal cord preparation using oblique infrared LED illumination; (right) epifluorescent and IR-LED images of a retrogradely labelled SPN. (**Ca**–**c**), characteristic discharges evoked by the saturating dorsal root stimulation (1 ms, 140 µA) in a low-output SPN (LO-SPN), a medium-output SPN (MO-SPN) and high-output SPNs (HO-SPNs). Here and in the following figures, the time moments of the stimulations are indicated by arrows. (**Da**), the histogram showing distribution of SPNs according to the number of spikes evoked by the dorsal root stimulation. (**Db**), a total number of spikes evoked in the populations of LO-, MO- and HO-SPNs by the saturating dorsal root stimulation.

Using a combination of fluorescent imaging (Fig. 1Ab) and the oblique IR-LED illumination technique (Fig. 1B) we visualized, in the isolated spinal cord preparation, lamina I SPNs for the tight-seal recordings (Fig. 1Aa). Based on their responses to the dorsal root stimulation at saturating strength (1 ms, 140 µA, 10 pulses at 0.1 Hz), SPNs could be divided in three groups. The largest group (n = 36) contained low-output SPNs (LO-SPNs) generating short-duration EPSPs and, on average, one spike (Fig. 1Ca,Da). The medium-output SPNs (MO-SPNs, n = 11) responded with bursts of three-to-six spikes (Fig. 1Cb,Da). A principally different type of response was observed in a group of high-output SPNs (HO-SPNs, n = 11), in which strong and prolonged EPSPs evoked firing of ten-to-twenty spikes (Fig. 1Cc,Da). Counting the total number of action potentials revealed that HO-SPNs, which represented only 19% of the SPN population, generated 69% of its output spiking activity (Fig. 1Db). Thus, the SPNs exhibit diverse output characteristics, and therefore, are likely to play different roles in nociceptive processing.

### Input-output characteristics of LO- and HO-SPNs

Three groups of SPNs showed principally different input-output characteristics in the experiments, in which dorsal roots were gradually stimulated to recruit Aδ- and C-fibers.

**Figure 2:**
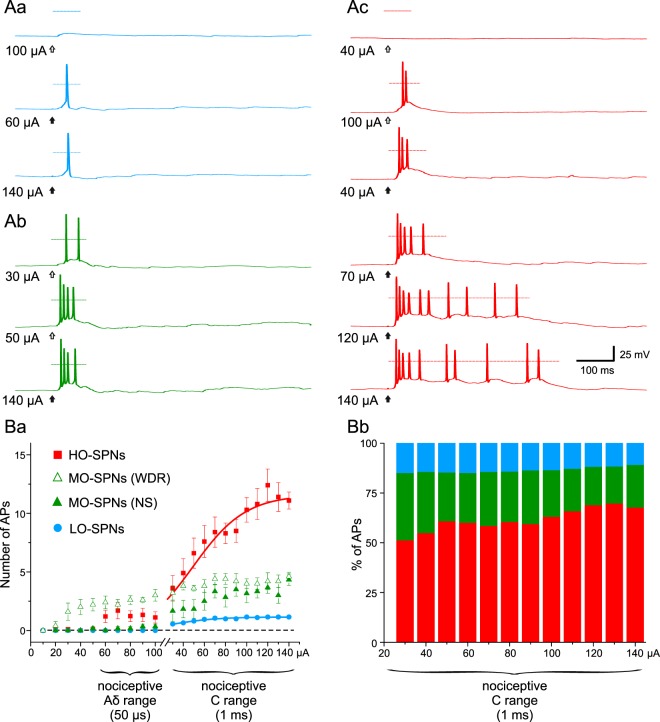
Input-output characteristics of SPNs. (**Aa**–**c**), spike discharges evoked in an LO-SPN, an MO-SPN and an HO-SPN by gradually stimulating dorsal roots. The strength of the current pulse is indicated near to the corresponding trace; open and filled arrows indicate 50 µs and 1 ms pulses, respectively. (**Ba**), the input-output characteristics of the LO-, MO-, and HO-SPNs. Each point represents the mean number of spikes evoked in each type of neuron at a given intensity of the dorsal root stimulation (WDR, wide dynamic range MO-SPNs; NS, nociceptive-specific MO-SPNs). (**Bb**), the relative contribution of the LO-, MO- and HO-SPNs to the total number of spikes generated by the entire population of SPNs as a function of the stimulation strength in the C-fiber range (1 ms).

LO-SPNs had firing threshold in the nociceptive C-fiber range (1 ms, 30–70 µA) and did not show more than one or, rarely, two spikes, even at the strongest stimulations tested (1.15 ± 0.04 spikes, n = 36; Fig. 2Aa). Thus, the LO-SPNs, responded in an all-or-nothing manner to the single high-threshold afferent stimulation.

In contrast to the other two groups, the MO-SPNs exhibited heterogeneous input-output characteristics. The majority of these neurons began to respond by single spikes or short bursts of spikes to innocuous (less than or equal to 50 μA, 50 µs; 5 of 11 cells) or noxious (more than 50 μA, 50 µs; 3 of 11 cells) Aδ-range stimulations (Fig. 2Ab). In 4 of MO-SPNs, a short discharge evoked by the Aδ-fiber stimulation was only slightly increased in the C-fiber range, indicating that these neurons preferentially processed an input from Aδ nociceptors. The remaining 7 MO-SPNs displayed a gradual increase in the number of spikes in the C-fiber range (4.4 ± 0.4 spikes at saturating stimulation of 150 μA, 1 ms). From a functional point of view, 5 MO-SPNs, responding to both innocuous and noxious stimuli, behaved as wide dynamic range neurons^11^, while the other 6 as nociceptive-specific neurons^8,9^ (Fig. 2Ba). Due to such a diversity in the input-output characteristics of this small group of neurons, the MO-SPNs were not further analyzed.

HO-SPNs began to discharge at the nociceptive Aδ-range stimulations (50 µs, 60–70 µA; 5 of 11 neurons) and the number of the spikes evoked further increased in the C-fiber range (Fig. 2Ac). Thus, HO-SPNs showed characteristics of nociceptive-processing neurons with a wide encoding range.

The functional difference between LO- and HO-SPNs became evident when comparing plots of their input-output characteristics (Fig. 2Ba). That for the LO-SPNs was simple and could be well fitted in the C-fiber range with a Boltzmann function (R^2^ = 0.99) reaching its saturation at a value close to one spike (1.15 ± 0.04, t-value = 28.02, p < 0.001; Fig. 2Ba). Thus, an LO-SPN is likely to simply transmit the nociceptive C-fiber input to the supraspinal structures. The characteristic for the HO-SPNs first showed a local saturation in the Aδ-fiber range (at 70–100 µA) but then increased in the nociceptive C-range (Fig. 2Ba). This increase could also be well approximated by a Boltzmann function (R^2^ = 0.98) showing saturation at 11.6 ± 0.7 spikes (t-value = 16.99, p < 0.001). This way, the HO-SPNs could detect the nociceptive Aδ-input and convert the strength of the C-fiber input to the increasing number of spikes. These data indicated distinct functional roles of the SPNs. From a physiological point of view, an LO-SPN simply relays the C-fiber nociceptive input, while an HO-SPN detects Aδ-input and encodes the strength of the C-fiber input.

The contribution of the HO-SPNs (n = 11) to the total number of spikes generated by the entire SPN population (n = 58) was above 50% in the whole C-fiber range (Fig. 2Bb), further emphasizing importance of this small group of neurons for nociceptive processing. Interestingly, the HO-SPN contribution to the overall output gradually increased with the C-fiber input (slope, 1.44 ± 0.19% per 10 µA, R^2^ = 0.86; Fig. 2Bb).

In the following experiments, we studied specific mechanisms underlying differential processing of the nociceptive afferent inputs by LO- and HO-SPNs.

### Different intrinsic properties of LO- and HO-SPNs

**Figure 3:**
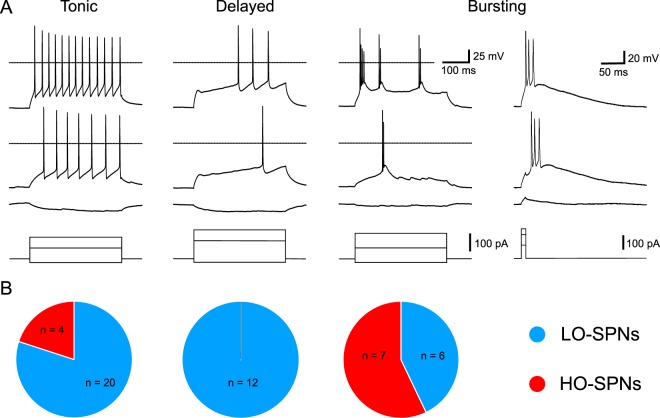
Intrinsic firing properties of LO-SPNs and HO-SPNs. (**A**), intrinsic firing in a tonic SPN, a delayed SPN and a bursting SPN elicited by the intracellular current injection (pulse duration, 500 ms). In the bursting SPN, short bursts of spikes were also evoked in an all-or-nothing manner by the intracellular injection of a short (10 ms) current pulse (right). (**B**), diagrams showing numbers of LO- and HO-SPNs among tonic, delayed and bursting neurons.

The resting membrane potentials and input resistances were not significantly different in LO-SPNs (−58 ± 1 mV and 0.73 ± 0.08 GΩ, n = 36) and HO-SPNs (−59 ± 1 mV, n = 11, p = 0.48; and 0.73 ± 0.20 GΩ, n = 11, p = 0.1). The membrane capacitance was however significantly larger in HO-SPNs than in LO-SPNs (93 ± 13 pF *versus* 63 ± 5 pF, p < 0.05). LO-SPNs showed tonic (n = 20), delayed (n = 12) and bursting (two spikes, n = 6) patterns of intrinsic firing, while HO-SPNs were tonic (n = 4) and bursting (n = 7; Fig. 3). In the last group, bursts of two-to-four spikes were also evoked in an all-or-nothing manner by short current injections (Fig. 3) or spontaneous synaptic activity (not shown). Statistical tests revealed a significant difference in the firing patterns of these neurons (p < 0.01, Fisher’s exact test). The HO-SPNs had significantly higher proportion of bursting neurons (3.8 times, p < 0.01, Fisher’s exact test with Bonferroni correction), which also responded with a short burst of spikes to afferent stimulations. Thus, difference in the intrinsic firing properties represents one of the mechanisms responsible for the higher number of spikes evoked in HO-SPNs by the primary afferent stimulation. Furthermore, intrinsic all-or-nothing bursts of spikes (Fig. 3A, right) allowed HO-SPNs to amplify their response to the nociceptive Aδ-input (Fig. 2Ac).

### LO- and HO-SPNs differ in their afferent supply

An analysis of the SPNs, which survived the entire experimental protocol (LO-SPNs, n = 30; HO-SPNs, n = 11), revealed a clear difference in organization of their afferent supply.

**Figure 4:**
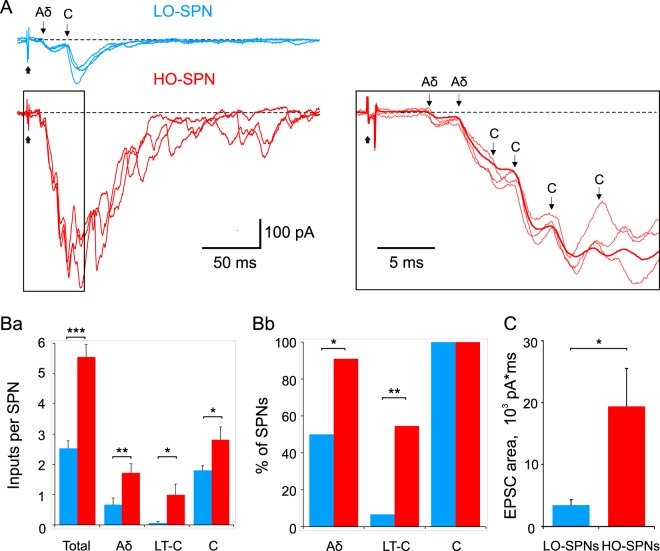
Monosynaptic primary afferent inputs. (**A**), monosynaptic EPSCs (indicated by arrows) mediated by Aδ- and C-afferents in an LO-SPN and an HO-SPN. Inset, three individual traces are shown superimposed with the average of 14 traces. Note that the HO-SPN has larger input with more numerous monosynaptic components. Holding potential, −70 mV. Root stimulation, 1 ms, 140 µA. (**Ba**), histogram showing the mean number of direct inputs per neuron for LO-SPNs (blue) and HO-SPNs (red). Here and in the following figures: *p < 0.05; **p < 0.01; ***p < 0.001. (**Bb**), percentage of LO-SPNs (blue) and HO-SPNs (red) receiving direct Aδ-, C- and low-threshold C- (LT-C) afferent inputs. (**C**), the strength of the primary afferent input calculated by integrating evoked EPSCs.

The number of all monosynaptic inputs per neuron in the population of HO-SPNs was 2.2 times higher than in LO-SPNs (p < 0.001; Fig. 4A and Ba). More specifically, HO-SPNs received higher number of direct inputs from Aδ- (2.6 times, p < 0.01), low-threshold C- (5.1 times, p < 0.05) as well as C-afferents (1.6 times, p < 0.05; Fig. 4Ba).

We also found a significant difference in a percentage of SPNs receiving each type of input (Fig. 4Bb). Although all SPNs in our sample were directly supplied by C-fibers, substantially higher percentage of HO-SPNs received monosynaptic Aδ-inputs (90.9% *versus* 46.7% for LO-SPNs, p < 0.05, Fisher’s exact test). Furthermore, more than a half of HO-SPNs (54.5%) received at least one low-threshold C-fiber input, whereas LO-SPNs were almost deprived of it (6.7%, p < 0.01, Fisher’s exact test).

To estimate the strength of the primary afferent inputs (both mono- and polysynaptic), we integrated the major EPSC component. At saturating C-fiber stimulations, the inputs to the HO-SPNs were 5.5 times stronger than those to the LO-SPNs (Fig. 4C; p < 0.05). Since the difference in the strength was substantially larger than that in the average number of direct inputs per a neuron (2.2 times), one could assume that HO-SPNs also received a stronger polysynaptic input.

Thus, the HO-SPNs receive a higher number of direct inputs from the nociceptive fibers and their overall primary afferent supply is substantially stronger than in LO-SPNs. Besides, the higher number of Aδ-inputs and the prevalence of the intrinsic bursting are two factors underlying ability of HO-SPNs to discharge in the nociceptive Aδ-fiber range, in which LO-SPNs did not generate spikes (Fig. 2Ac and Ba). Furthermore, a combination of a strong afferent supply and intrinsic bursting could explain an intensive initial discharge evoked in the HO-SPNs by C-fiber-range stimulation.

### HO-SPNs, but not LO-SPNs, integrate network activity

In contrast to LO-SPNs, the HO-SPNs continued firing for hundreds of milliseconds after arrival of afferent volley (see Figs. 1 and 2). In the following experiments, we studied cellular mechanisms underlying the prolonged late firing.

**Figure 5:**
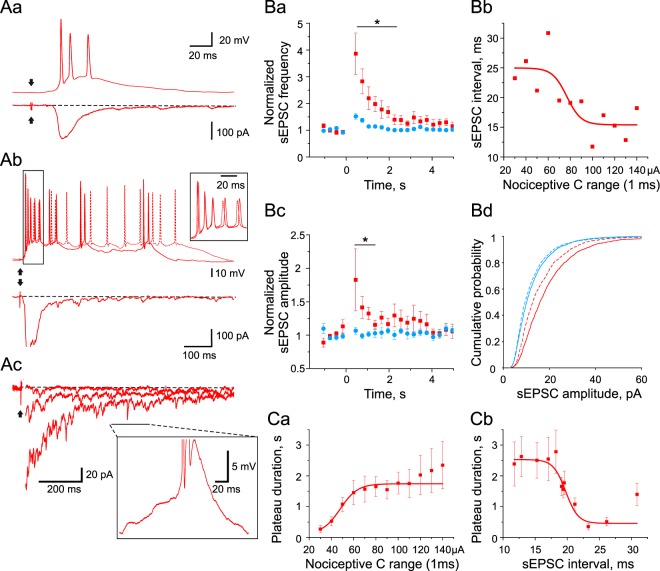
HO-SPNs integrate the network activity. (**Aa**), a weak stimulation (50 µs, 70 µA) of the dorsal root evoked in an HO-SPN an initial discharge of several spikes. (**Ab**), a strong stimulation (1 ms, 130 µA) evoked the initial discharge followed by a plateau potential and a late discharge. Two superimposed traces demonstrate that spikes in the initial discharge are time-locked (an inset), whereas spikes in the late discharge are randomly generated during the plateau potential. Note that the firing continued after the major component of the afferent input terminated (voltage-clamp trace). **Ac**, voltage-clamp recordings at higher amplification revealed an appearance of the slow inward current and numerous spontaneous EPSCs (sEPSCs). The dorsal root was stimulated at increasing strength (20 µA, 40 µA and 80 µA, 1 ms). The major component of the primary afferent input is truncated. Inset, an example of current-clamp recording from the same neuron after the strong stimulation during a time interval indicated by a line. Note, a spike triggered by the summation of sEPSPs during the plateau potential. Recordings in **Aa-c** are from one and the same HO-SPN. (**Ba**), changes in the frequency of sEPSCs in LO-SPNs (blue) and HO-SPNs (red) after the dorsal root stimulation (time, 0) at saturating C-fiber strengths. (**Bb**), the interval between sEPSCs in HO-SPNs as a function of the strength of the dorsal root stimulation. (**Bc**), the time course of the changes in the amplitudes of sEPSCs in LO-SPNs (blue) and HO-SPNs (red) after the dorsal root stimulation (time, 0). (**Bd**), a transient increase in the sEPSC amplitude in HO-SPNs, but not in LO-SPNs, during the first 0.9 s after the root stimulation. (**Ca**), the plateau potential duration as a function of the dorsal root stimulation strength. (**Cb**), an increase in duration of the plateau potential correlated with a reduction in the intervals between sEPSCs.

At weak suprathreshold stimulations (10–30 μA above the threshold), the mono- and polysynaptic EPSPs in HO-SPNs evoked an initial discharge of several spikes resembling the pattern of intrinsic firing in these neurons (Fig. 5Aa). At stronger stimulations, the initial discharge (Fig. 5Ab, inset), was followed by a plateau potential and concomitant late discharge (Fig. 5Ab, current-clamp trace), which continued after termination of the major component of the afferent input (Fig. 5Ab, voltage-clamp trace). Interestingly, the quantitative analysis has shown that the late phase of discharge made a considerable contribution to the HO-SPN output (7.6 ± 2.0 *versus* 5.2 ± 1.5 spikes, initial *versus* late discharge at saturating afferent stimulation). Examination of the voltage-clamp traces revealed an appearance of a small (5–50 pA) slowly-decaying (hundreds of milliseconds to seconds) inward current and numerous spontaneous EPSCs (sEPSCs) (Fig. 5Ac). In HO-SPNs, having an input resistance of 0.73 ± 0.20 GΩ, this slowly-decaying current was sufficient to generate a depolarizing plateau potential of up to 20 mV (Fig. 5Ab). During the plateau potential, sEPSPs started to trigger random spikes (Fig. 5Ac, inset), what rarely happened at the resting membrane potential (6.4 s^−1^ during the plateau *versus* 0.4 s^−1^ at the resting membrane potential, p < 0.001).

To better understand the contribution of spontaneous events to the late discharge, we analyzed them in a time frame from 0.3 s to 5.0 s after the root stimulation, when the monosynaptic and major component of polysynaptic afferent input terminated. In HO-SPNs, the sEPSC frequency at the beginning of the frame increased by a factor of 3.9 ± 0.8 (n = 7, p < 0.05) and returned to its basal level within 2 s (Fig. 5Ba). This effect was specific for HO-SPNs, as in LO-SPNs it was negligible. The median inter-event intervals in the HO-SPNs decreased with the stimulation intensity (R^2^ = 0.66 for Boltzmann function, lower bound: 24.95 ± 2.22 ms, t-value = 11.24, p < 0.001; upper bound: 15.41 ± 1.77 ms, t-value = 8.72, p < 0.001; Fig. 5Bb), indicating a gradual activation of the intrinsic neuronal circuitry, targeting this population of SPNs. Furthermore, we observed a transient increase in the sEPSC amplitude in HO-SPNs, but not in LO-SPNs, during the first second of the time frame indicating action potential-dependent mechanism of this increase (Fig. 5Bc and Bd). This indicated that a specific increase in the frequency of high-amplitude sEPSPs induced by reverberating network activity contributed to the induction of the late firing in the HO-SPNs (Fig. 5Ab, Ac inset). Such observations imply that the HO-SPNs are specifically supplied by a dorsal horn circuitry responsible for their late firing.

Our data also provide evidence that the rise in the spontaneous network activity is synchronized with the generation of the intrinsic plateau potential to increase discharges in the HO-SPNs. First, the plateau potential and the increase in the frequency and amplitude of sEPSCs showed similar time courses (Fig. 5Ba, Bc and Ca). Second, duration of the plateau potential also increased with the stimulation strength (R^2^ = 0.98 for Boltzmann function; upper bound: 1.74 ± 0.08 s, t-value = 22.68, p < 0.001; Fig. 5Ca). Third, an increase in duration of the plateau potential correlated with reduction in the inter-event intervals for the spontaneous activity (Spearman correlation coefficient, −0.81, p < 0.001; Fig. 5Cb). This way, the plateau potential created a ‘window’ for a dramatic (16 fold) increase in the probability of spontaneous events to reach the threshold of action potential firing.

Thus, the late discharge was driven by the plateau potential and activation of the local neuronal network. These factors increased with intensity of stimulation leading to enhancement of the late discharge.

### Signal processing in HO-SPNs is NMDA-receptor-dependent

**Figure 6:**
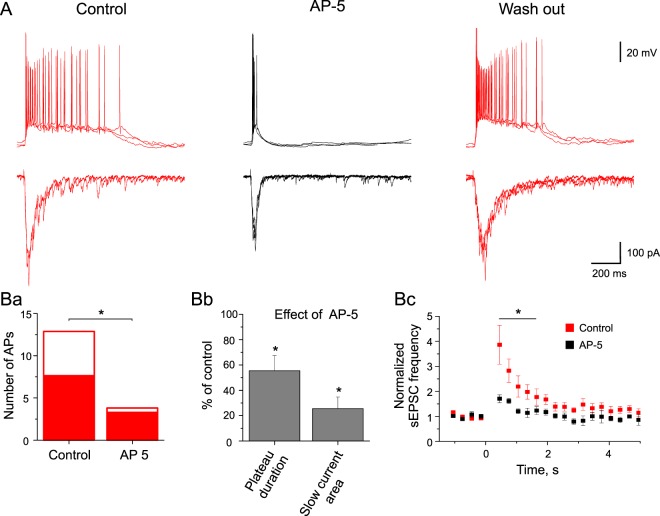
Effects of the NMDA receptor blocker on the excitability of HO-SPNs. (**A**), AP-5 at 40 µM reversibly suppressed the late phase of discharge in an HO-SPN. Note the shortening of the plateau potential and inhibition of the slow inward current (voltage-clamp traces). Effects of AP-5 on: (**Ba**), the number of spikes generated in the initial burst (filled) and during the late discharge (open); (**Bb**), the plateau potential duration and the slow inward current magnitude (calculated as area under the curve); (**Bc**), the frequency of sEPSCs evoked by the root stimulation.

NMDA receptors are expressed at primary afferent synapses on SPNs and also play an important role in a polysynaptic drive to these neurons^13,14^. To study whether the signal processing in the HO-SPNs depends on NMDA receptors, we analyzed effects of their specific blocker AP-5. The bath application of the blocker reversibly suppressed the late phase of discharge (0.5 ± 0.5 spikes in AP-5 *versus* 5.2 ± 1.5 spikes in control, n = 4, p < 0.05; Fig. 6A and Ba), whereas the initial burst of spikes was less affected (3.3 ± 1.5 spikes in AP-5 *versus* 7.6 ± 2.0 spikes in control, n = 4, p < 0.05; Fig. 6A and Ba). At the same time, we observed a significant shortening of the plateau potential (to 55 ± 12%, n = 4, p < 0.05) and an inhibition of the slow inward current generating this plateau (to 25 ± 9%, p < 0.05, n = 4; Fig. 6A and Bb). Furthermore, the stimulation-induced increase in the sEPSC frequency was suppressed by the NMDA receptor block (n = 4, p < 0.05; Fig. 6Bc) due to the inhibition of the local neuronal circuitry targeting these SPNs. Thus, it could be concluded that the NMDA-receptor-dependent processes contribute to the late phase of discharge and play a crucial role in the signal processing by HO-SPNs.

## Discussion

We used the *ex-vivo* spinal cord preparation with an attached dorsal root to record from SPNs, which preserved their dendritic structure, primary afferent inputs and synaptic supply from the intrinsic spinal circuitries. This approach allowed to identify distinct populations of the SPNs and their specific mechanisms of the nociceptive input processing.

### Afferent inputs to LO- and HO-SPNs

A modern view of the signal processing in lamina I SPNs is mainly based on *in vitro* recordings performed in thin slice preparations of the spinal cord. These studies show that only a minority of lamina I SPNs receive direct inputs from Aδ- and C-afferents (9% and 32% of SPNs, respectively)^15^. More recent reports further assume that several classes of nociceptors do not excite SPNs directly but do this via activation of specific populations of dorsal horn interneurons^16,17^. Here we used a preparation which preserved both the central terminals of primary afferents and the dendritic structure of the receiving spinal neurons^18–21^, to carry out a detailed analysis of the afferent supply of lamina I SPNs. In our experiments, all the neurons received monosynaptic C-fiber input and approximately a half of them additionally showed direct Aδ-fiber input. Such strong supply of SPNs by nociceptive fibers is consistent with immunocytochemical studies demonstrating a high number of direct contacts formed by Aδ- and C-afferents with the somata and proximal dendrites of lamina I SPNs^22–24^. From a functional point of view, these suprathreshold Aδ/C-fiber inputs allow SPNs to directly transmit information about the noxious stimuli.

**Figure 7:**
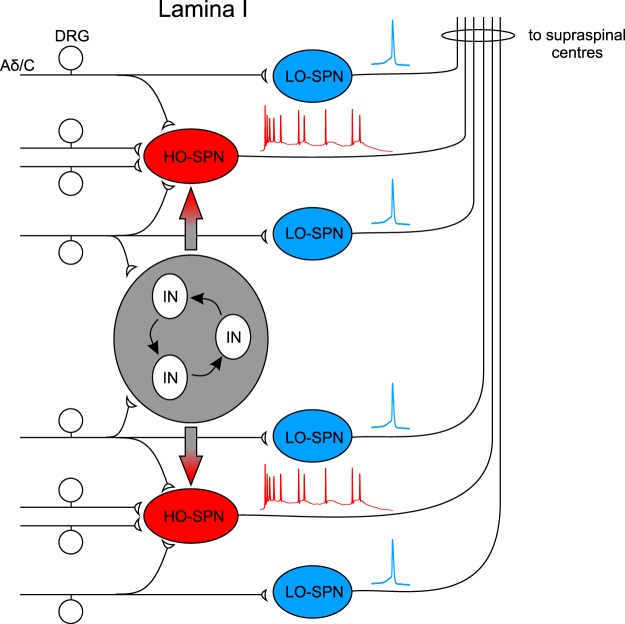
Putative scheme of the signal processing by lamina I SPNs. More numerous LO-SPNs receive supply from few primary afferents and may function as direct transducers of the high-threshold nociceptive stimuli. The signal processing in LO-SPNs is not strongly influenced by the intrinsic spinal circuitries. Individual LO-SPNs use a single spike code to transmit information about threshold and location of the noxious stimulus. Less numerous HO-SPNs receive direct inputs from a larger number of nociceptive afferents as well as from the intrinsic spinal circuitry. The HO-SPNs can gradually encode intensity of noxious input and generate the major part of the spiking activity send to the supraspinal centres.

Responses of supraspinally projecting neurons to single transcutaneous electrical stimulation were studied using *in vivo* extracellular recordings in rats, mice and cats^8–12^. These experiments identified early and late spike discharges, which were characterized as mediated by peripheral Aδ- and C-fibers, respectively. Although early and late discharges were also observed in our experiments, detailed analysis of patch clamp recordings of synaptic input underlying action potential output suggested another explanation. The early discharge was induced by the monosynaptic Aδ- and C-fiber inputs amplified by intrinsic firing properties of a neuron. In contrast, the late discharge lasting for seconds after a single stimulation was generated by the network-driven reverberating activity in the circuitry supplying an HO-SPN (Fig. 7).

### Mechanisms of signal processing in SPNs

Mechanisms of signal processing in LO-SPNs and HO-SPNs are principally different. LO-SPNs received a direct input from a few C- and Aδ-fibers and generate an output mainly consisting of one spike; interestingly, the monosynaptic Aδ-fiber input was always subthreshold. Furthermore, these cells are weakly involved in the local neuronal network, since even saturating stimuli produced only a minor increase in their synaptic drive, which did not evoke additional spikes. In comparison with the LO-SPNs, the HO-SPNs received more numerous and stronger monosynaptic inputs from Aδ- and C- fibers. These inputs amplified by the intrinsic firing properties were responsible for generation of early discharges. This amplification was especially important for conversion of the Aδ-nociceptive input into a short burst of spikes. Besides, the stronger input and higher incidence of intrinsic bursting underlie a gradual increase in the number of spikes in the early discharge in the C-fiber stimulation range.

The late component of the discharge seen in an HO-SPN was caused by a slow NMDA-receptor-dependent current and long-lasting network activity, rather than by mono- or polysynaptic inputs. The slow current generated a plateau potential facilitating late spiking triggered by the network activity. Both the plateau potential duration and evoked network activity increased with a strength of the C-fiber stimulation. Thus, the plateau potential created a time window, in which an increase in the network activity was converted into the number of spikes constituting the late discharge. As our data show, this network activity is generated by a specific neuronal circuitry targeting HO-SPNs rather than LO-SPNs. Such circuity may be formed by both the superficial and deep dorsal horn interneurons firing similar long-lasting discharges in response to the afferent stimulation at C-fiber strength^25–27^.

### Encoding of nociceptive input

Here we show that the lamina I SPNs can be divided in at least two distinct groups, the LO- and HO-SPNs (Fig. 7). The LO-SPNs, representing a majority of the SPNs, generate one spike in response to the single suprathreshold C-range stimulation. Thus, they simply transmit the primary afferent activity to the brain and are likely to function as nociceptive input transducers. They receive input from a few primary afferents what may reflect the fact of having small peripheral receptive fields as shown for lamina I cells in *in vivo* studies^28^. These properties may be important for encoding location of the noxious stimulus. In agreement with this, the output of the LO-SPNs is not affected by the afferent-driven activity in the intrinsic spinal circuitries. Thus, the LO-SPNs are good candidates for relaying information about the site of noxious stimulus.

In contrast, HO-SPNs integrate direct inputs from a larger number of nociceptive afferents and intrinsic spinal circuitry (Fig. 7) and are, therefore, less suitable for encoding precise stimulus location. Instead, the HO-SPNs can gradually encode the noxious stimulus intensity. In a broad range of the C-fiber stimulations, the strength of the nociceptive input is converted to the number of spikes. Some HO-SPNs also use a short burst code to transmit information about activation of the nociceptive Aδ-input. The overall importance of the HO-SPNs for the nociceptive processing is emphasized by the fact that these neurons generate the major part of the lamina I output activity sent to the brain.

It is possible that the early and late components of HO-SPN output encode different aspects of nociceptive input. The early component, directly evoked by the afferent volley, is gradually increased with intensity of the dorsal root stimulation in the C-fiber range (from 2 to 8 spikes). This way, the early component encodes the quantitative aspect of the mono- and polysynaptic afferent supply of a particular HO-SPN. In contrast, the late discharge reflects the stimulation-induced long-lasting activity in the dorsal horn network. This activity is triggered by the broad primary afferent input to this specific network. Thus, the late discharge is likely to encode a total number of nociceptive C-fibers supplying this network. The temporal patterns of early and late discharges are also different. Spikes in the former are mostly time-locked, have high frequency and are generated within 200 ms after the stimulation, while the latter are not time-locked, have lower frequency and last for seconds after stimulation. Thus, an HO-SPN generating the early and late discharges may use distinct temporal codes to transmit, via the same ascending axon, different aspects of nociceptive information.

### Functional implications

Our results may also have some implications for the functional roles of the LO- and HO-SPNs in processing different aspects of pain. These neurons target parabrachial area, which, in turn, projects to the amygdala, the region involved in processing emotional aspect of pain^3,6,7^. The emotional component can be encoded in two ways. First, by individual HO-SPNs generating increasing number of spikes in a broad range of noxious stimulation. The second possibility arises from the fact that receptive fields of parabrachial neurons are much larger than those of lamina I SPNs, indicating a large convergence of the latter onto former. In this case, gradual recruitment of LO-SPNs converging onto one parabrachial target may also encode the strength of the emotional component of pain. However, individual LO-SPNs are unlikely to encode the emotional component of pain.

On the other hand, individual lamina I neurons project to more than one supraspinal area^1,5,29^, and those targeting parabrachial area can also project to the thalamus^2,5,29–31^. The spinothalamic pathway projecting to the primary somatosensory cortex is involved in processing discriminative aspects of pain^6,32^. In the context of discriminative processing, the LO-SPNs may localize the stimulus, while the HO-SPNs may encode its strength.

Thus, two groups of SPNs are likely to play different roles in nociception functioning as transducers or intensity encoders of peripheral input. They may be also involved in encoding the ascending signals, describing emotional as well as discriminative aspects of pain. Furthermore, specific reduction in activity or silencing of the small population of HO-SPNs may substantially attenuate transmission of nociceptive signals to the brain; these neurons may represent an adequate target for treating acute pain without aversive side effects on other sensory modalities.

## Materials and Methods

### Ethical approval

All experimental procedures were in accordance with the European Commission Directive (86/609/EEC) and ethical guidelines of the International Association for the Study of Pain and were approved by the local Animal Ethics Committee of the Bogomoletz Institute of Physiology (Kiev, Ukraine).

### SPN labelling

For retrograde labelling, fluorescent dye aminostilbamidine (an analogue of Fluoro-Gold tracer) was injected to the parabrachial area in the brainstem, which is targeted by a vast majority of lamina I SPNs^1,30^. Since individual neurons project to more than one target^1^, the population of SPNs labelled from the parabrachial area also contained those projecting to other regions including thalamus and periaqueductal gray matter^5,30^.

A Wistar rat (P20-P21) was anesthetized with a mixture of ketamine (65 mg/kg) and xylazine (10 mg/kg). The animal head was fixed in a stereotaxic apparatus and a rostrocaudal incision of skin was performed along the midline to expose bregma and occipital bone. Aminostilbamidine (200 nl, 2%) was injected using a 1-µl Neuros syringe with 32-gauge needle (Hamilton, USA) at the following stereotaxic coordinates (with respect to bregma): 13.2 mm rostrocaudal, 2 mm mediolateral and 5.6 mm dorsoventral (angle 30^°^). After the needle withdrawal, the skin was sutured and treated with Betadine (Egis, Hungary). The animals were kept on a warm pad (30 °C) during the whole procedure and until full recovery from anesthesia. Three-four days were given for retrograde transport of the dye to the somata of SPNs.

### Experimental design

#### *Ex vivo* spinal cord preparation

A rat (P24-P28) was killed by decapitation and the vertebral column cut out and immersed at 20–22 °C into oxygenated sucrose solution containing (in mM): 200 sucrose, 2 KCl, 1.2 NaH_2_PO_4_, 0.5 CaCl_2_, 7 MgCl_2_, 26 NaHCO_3_, 11 glucose (pH 7.4 when bubbled with 95% O_2_ and 5% CO_2_). The spinal cord was gently removed with attached L5 or L5 and L4 dorsal roots cut close to the corresponding ganglia. The lumbar spinal cord was cleaned from the dura and pia matter, to provide access for the recording pipette, glued with cyanoacrylate adhesive to a metal plate and transferred to the recording chamber. Neurons in the region between the dorsolateral funiculus and the dorsal root entry zone^33^ were visualized using the oblique infrared light-emitting-diode (IR-LED) illumination technique^34,35^. Specific equipment used for implementation of this technique and for acquisition of fluorescent images was recently described in detail^36^.

#### Electrophysiological recordings

The whole-cell recordings from labelled lamina I SPNs in the L4-L5 segments (Fig. 1Aa) were performed at room temperature (20–22 °C) in oxygenated artificial cerebro-spinal fluid containing (in mM): NaCl 125, KCl 2.5, CaCl_2_ 2, MgCl_2_ 1, NaH_2_PO_4_ 1.25, NaHCO_3_ 26 and glucose 10 (pH 7.4, 95% O_2_ and 5% CO_2_). Patch pipettes were pulled from borosilicate glass using a P-87 horizontal puller (Sutter Instruments, USA); Pipette resistances were 3–5 MΩ when filled with intracellular solution containing (in mM): 145 K-gluconate, 2.5 MgCl_2_, 10 HEPES, 2 Na_2_-ATP, 0.5 Na-GTP and 0.5 EGTA (pH 7.3). Signals were acquired and filtered at 2.6 kHz using MultiClamp 700B amplifier (Molecular Devices, CA, USA) and digitized at 10 kHz with Digidata 1320 A under control of pClamp 9.2 software (Molecular Devices, CA, USA). Offset potentials were compensated before seal formation. Liquid junction potentials were not compensated.

#### Dorsal root stimulations

Dorsal roots (L4 or L5) were stimulated (Fig. 1Aa) via a suction electrode using ISO-Flex (AMPI, Israel) stimulator^36,37^. A 50 µs pulse of increasing amplitude (10–100 µA) was applied to recruit Aβ- and Aδ-fibers and a 1 ms pulse (30–140 µA) to activate all fibers, including high-threshold Aδ- and C-afferents. To avoid wind-up, observed in spino-parabrachial neurons^38^, stimulation frequency was set at 0.1 Hz. Monosynaptic excitatory postsynaptic currents (EPSCs) were identified on the basis of low failure rates and small latency variations as described previously^33,39^. The afferent conduction velocity (CV) was calculated by dividing the conduction distance by the conduction time. The former included the length of the root from the opening of the suction electrode to the dorsal root entry zone and the estimated pathway within the spinal cord. The spinal pathway was measured from video images and calculated as the sum of the rostrocaudal and mediolateral distances between the cell body and the corresponding dorsal root entry zone. The conduction time was calculated for a monosynaptic EPSC from its latency with a 1 ms allowance for synaptic transmission.

Fibers mediating monosynaptic inputs to lamina I SPNs were classified based on criteria developed by Fernandes *et al*.^27^. Aδ-fibers: activated by a 50 µs pulse, CV > 0.5 m/s; C-fibers: activated by a 1 ms pulse, CV < 0.5 m/s^40^. The afferents with Aδ-fiber CVs activated only by a 1 ms pulse stimulation were classified as high-threshold Aδ. For simplicity, Aδ- and the high-threshold Aδ-afferents were analyzed as one group. The fibers were considered as low-threshold C afferents if activated by a 50 µs pulse stimulation and had CVs < 0.5 m/s.

### Data analysis

The data were analyzed using Clampfit 9.2 software (Molecular Devices, USA). Spontaneous EPSCs (sEPSCs) were detected and analyzed using MiniAnalysis software (Synaptosoft, USA) as described previously^41,42^.

For analysis, evoked EPSCs in HO-SPNs were divided into major and slow components. The transition between these two components was determined using voltage-clamp recordings at a time point where the EPSC decayed to 10% of its peak value.

### Statistical analysis

Unless otherwise stated, data sets were compared using Student’s t-test and all numbers are given as mean ± SEM. All trends were analyzed for significance using t-statistics for regression. The *p* < 0.05 was considered as statistically significant.

### Chemicals and Drugs

AP-5 was from Tocris (UK). Aminostilbamidine was supplied by Thermo Fisher Scientific (USA). Ketamine was from Farmak (Ukraine) and xylazine was from Biovet Pulawi (Poland). All other chemicals were obtained from Sigma-Aldrich (USA).
